# Ultra-Processed Food Consumption and Adult Diabetes Risk: A Systematic Review and Dose-Response Meta-Analysis

**DOI:** 10.3390/nu13124410

**Published:** 2021-12-09

**Authors:** Sajjad Moradi, Mohammad ali Hojjati Kermani, Reza Bagheri, Hamed Mohammadi, Ahmad Jayedi, Melissa M. Lane, Omid Asbaghi, Sanaz Mehrabani, Katsuhiko Suzuki

**Affiliations:** 1 Halal Research Center of IRI, FDA, Tehran 314715311, Iran; sajadmoradi9096@gmail.com; 2 Nutritional Sciences Department, School of Nutritional Sciences and Food Technology, Kermanshah University of Medical Sciences, Kermanshah 6715847141, Iran; 3 Clinical Tuberculosis and Epidemiology Research Center, National Research Institute of Tuberculosis and Lung Diseases (NRITLD), Masih Daneshvari Hospital, Shahid Beheshti University of Medical Sciences, Tehran 14167-53955, Iran; IMHOJJATI@gmail.com; 4 Department of Exercise Physiology, University of Isfahan, Isfahan 81746-73441, Iran; will.fivb@yahoo.com; 5 Department of Clinical Nutrition, School of Nutritional Sciences and Dietetics, Tehran University of Medical Sciences, Tehran 14176-13151, Iran; mohamadihd@gmail.com; 6 Department of Community Nutrition, School of Nutritional Science and Dietetics, Tehran University of Medical Sciences, Tehran 14176-13151, Iran; ahmadjayedi@yahoo.com; 7 IMPACT (The Institute for Mental and Physical Health and Clinical Translation), Food & Mood Centre, School of Medicine, Barwon Health, Deakin University, Geelong, VIC 3217, Australia; Melissa.Lane@deakin.edu.au; 8 Cancer Research Center, Shahid Beheshti University of Medical Sciences, Tehran 14167-53955, Iran; omid.asbaghi@gmail.com; 9 Department of Clinical Nutrition, School of Nutrition and Food Science, Isfahan University of Medical Sciences, Isfahan 81746-73461, Iran; 10 Faculty of Sport Sciences, Waseda University, 2-579-15 Mikajima, Tokorozawa 359-1192, Japan

**Keywords:** ultra-processed food, diabetes risk, meta-analysis

## Abstract

(1) Background: Recent individual studies have demonstrated that consumption of ultra-processed food (UPF) may be related to type two diabetes mellitus (T2DM). We aimed to synthesize the results from these individual studies by conducting an updated systematic review and meta-analysis of observational studies evaluating the association between UPF consumption and the risk of T2DM. (2) Methods: A systematic search was conducted using ISI Web of Science, PubMed/MEDLINE and Scopus electronic databases from inception up to August 2021. Data were extracted from five studies (one cross-sectional study and four cohort studies, totaling 230,526 adults from four different countries). Risk ratios (RR) of pooled results were estimated using a random-effects model. (3) Results: Our results revealed that higher UPF consumption was significantly associated with an increased risk of T2DM (RR = 1.74; 95% CI: 1.36, 2.22; I^2^ = 68.9%; *p* < 0.001; *n* = 5). Linear dose-response analysis indicated that each 10% increase in UPF consumption (kcal/d) was associated with a 15% higher risk of T2DM (RR = 1.15; 95% CI: 1.06, 1.26; I^2^ = 86.0%; *p* < 0.001; *n* = 5) among adults. Non-linear dose-response analysis demonstrated a positive linear association between UPF consumption and T2DM (*p*_nonlinearity_ = 0.13, *p*_dose-response_ < 0.001; *n* = 5) among adults. (4) Conclusions: A higher intake of UPF was significantly associated with an increased risk of T2DM. However, underlying mechanisms remain unknown and future experimental studies are warranted.

## 1. Introduction

The growing incidence of chronic non-communicable diseases such as type two diabetes mellitus (T2DM) is the major healthcare concern worldwide [1]. According to reports from the International Diabetes Federation (IDF), approximately 6.28% (451 million) of the world population had T2DM in 2017, which resulted in more than 1 million deaths per year [2]. The prevalence of diabetes is projected to increase in the coming years, reaching 7079 and 7862 per 100,000 people in 2030 and 2040, respectively [3]. Across the globe, T2DM is one of the main causes of disability and reduced life expectancy [4]. Given the role of T2DM in the global burden of disease, there is an urgent need to investigate and address possible contributors to this disease, including modifiable lifestyle factors such as poor diet quality. Poor diet quality is characterized by low intakes of fruits, vegetables, whole grains, nuts and legumes and high intakes of added sugar, processed meats, refined grains and fried foods, which increase the risk of T2DM [5].

In the last half-century, the processing of foodstuffs has substantially evolved [6]. The NOVA food classification system, developed in 2009 by researchers from the University of São Paulo, is a relatively novel tool that categorizes foods based on the degree of processing. According to NOVA, foods and food products are classified into four groups, unprocessed or minimally processed food, processed culinary ingredients, processed food and ultra-processed foods (UPF). UPF is industrial formulations generated through compounds extracted, derived, or synthesized from food or food components [6,7,8]. They typically contain five or more ingredients made from high-yield food substances, including ingredients rarely used in the kitchen such as hydrogenated oil and fructose corn syrup and artificial food additives including sweeteners and colors emulsifiers and preservatives [6,7,8]. The products are inexpensive and shelf-stable formulations that are virtually imperishable, easily consumable and highly pleasurable in terms of their sensory properties; these are formulations that are ultimately displacing non-UPF [9,10]. UPF also typically contain nutrient profiles that are implicated in the risk of T2DM, such as high amounts of energy, trans- and saturated fat, sugar and salt, coupled with low levels of fiber and nutrient densities compared to non-UPF [6,8,11,12,13,14,15]. Concerns exist because of a global and upward trend in recent decades in the consumption of UPF in place of non-UPF, with a recent systematic review and meta-analysis of observational studies showing that UPF accounted for up to half of total daily energy [16]. Indeed, this review also demonstrated that increased consumption of UPF was associated with a higher risk of more than 10 chronic non-communicable diseases and premature death [16]. However, a meta-analysis on the association between UPF intake and risk of T2DM was not possible at the time of this review’s publication, due to a limited number of studies (*n* = 1). Since then, several individual studies have assessed this association. Therefore, the present study aimed to build on previous work by synthesizing the findings of observational studies investigating the association between UPF intake and the risk of T2DM.

## 2. Materials and Methods

The current systematic review and meta-analysis were conducted based on the 2020 PRISMA guidelines [17]. The study protocol was submitted and approved in the international prospective register of systematic reviews database (PROSPERO) under the registration number: CRD42021273097.

### 2.1. Literature Search and Selection

A systematic search was conducted using the following electronic databases: ISI Web of Science, PubMed/MEDLINE and Scopus, from database inception up to 10 August 2021 without restrictions for language. Search terms were a combination of free-text terms and controlled vocabulary related to UPF and diabetes, including the following that were applicable to a search via MELINE: ((“fast foods” [All Fields] OR “fast foods” [MeSH Terms] OR “ultra processed food *” [All Fields] OR “ultraprocessed food *” [All Fields] OR “ultra processed food *” [All Fields] OR “processed food *” [All Fields] OR “ultra-processed” [All Fields] OR “ultraprocessed” [All Fields] OR “ultra-processed” [All Fields] OR “NOVA” [All Fields] OR “nova food classif *” [All Fields] OR “nova food *” [All Fields] OR “nova food classif *” [All Fields] OR “NOVA food classification system” [All Fields]) AND (“Diabetes Mellitus” [MeSH Terms] OR “diabetes mellitus, type 2” [MeSH Terms] OR “Diabetes Mellitus” [All Fields] OR “diabetes” [All Fields] OR “T2DM” [All Fields] OR “type 2 diabetes mellitus” [All Fields])) (see Supplementary Table S2 for search terms used across the varied databases). The search strategy for grey literature consisted of a manual search of all original articles cited in the retrieved review articles.

### 2.2. Inclusion and Exclusion Criteria

The inclusion criteria consisted of the following: observational studies (cohort, case–control, or cross-sectional studies) undertaken in adults (≥18 years) that reported on the association between UPF consumption and the risk of T2DM, and provided effect estimates in the form of hazard ratio (HR), relative risk (RR), or odds ratios (OR) with 95% Confidence Interval (95% CI). Studies conducted in children and adolescents (<18 years), reviews, conference letters, notes, reports, short surveys and case reports were excluded. The population, intervention, comparator and outcome (PICO) description can be observed in Supplementary Table S1.

### 2.3. Study Selection

The assessment of titles and abstracts and the full-text review process for studies retrieved through our search strategy were undertaken separately by two researchers (S.M. and H.M.), with any disagreements about the inclusion and exclusion of selected studies decided by consensus or discussion. A standardized method was applied to the inclusion and exclusion criteria, which took into consideration the setting, population and evaluated exposure(s) and outcome(s) of individual studies.

### 2.4. Data Extraction

A standardized method was also applied to the data extraction process undertaken independently by two researchers (S.M. and H.M.) through Microsoft Office Excel 2013 (Microsoft Corporation, Redmond, WA, USA). The following parameters were extracted: (a) the first author’s name; (b) year of publication; (c) country and setting of the study; (d) number of participants, (e) age, (f) gender; (h) follow-up duration in cohort studies; (i) methods for evaluating exposure; (j) study main findings; and (k) covariates applied for adjustments in the multivariable analyses. Any discrepancies and disagreements about data extraction were determined by consensus or discussion with a third researcher (A.J.).

### 2.5. Quality Assessment

Two researchers (S.M. and H.M.) independently evaluated the quality of each study by applying the Newcastle–Ottawa Scale (NOS) [18]. The NOS was designed to examine the quality of non-randomized studies to fit for meta-analyses and assigns a maximum of nine points for the least risk of bias in three broad domains: Study group selection (four points); study group comparability (two points); and exposure and outcome ascertainment for case–control or cohort studies, respectively (three points). Disagreements that were resolved by the consensus outcome of the quality assessment for each study are reported in Table 1.

### 2.6. Data Synthesis and Statistical Analyses

We conducted statistical analyses with STATA version 14.0 (StataCorp, College Station, Lakeway, TX, USA) and SPSS version 25.0 (IBM, Armonk, NY, USA). The OR and its 95% CI were assumed as the effect size. The effect estimates reported by the original studies and considered for inclusion in our meta-analyses included OR and HR (and their 95% CI); HR was considered equal to RR [23]. The synthesized effect estimates for the current study were expressed as pooled OR with 95% CI. Due to anticipated heterogeneity between studies, the effect estimates were calculated using a weighted random-effects model using the DerSimonian–Laird approach [24]. First, we conducted a pairwise meta-analysis by combining the effect sizes for the highest compared with the lowest category of UPF consumption. Heterogeneity among the studies was examined by the Cochran Q and I-squared (I^2^) statistics. The I^2^ value was calculated as ([Q-df])/Q × 100%, Q being the χ^2^ value and df the corresponding degrees of freedom. The heterogeneity was considered significant where the Q statistics were significant (*p* < 0.01) or I^2^ > 50%; more specifically, low, moderate, high and extreme heterogeneity was defined according to the I^2^ statistics cut-offs of <25%, 25–50%, 50–75% and ˃75%, respectively. Sensitivity analysis was carried out by removing each study and recalculating the pooled effect estimates (i.e., one study-removed analysis). Publication bias was assessed by the visual inspection of funnel plots, formal testing by the Egger’s regression asymmetry and Begg’s rank correlation tests [25,26] and results were considered significant at *p* < 0.05.

We also conducted a dose-response meta-analysis to estimate the RRs for each 10% increment in UPF intake, according to the method introduced by Greenland and Orsini [27,28]. For this purpose, studies needed to report the number of cases and non-cases or person-years and median point of UPF across more than three categories of UPF consumption. Finally, we performed a one-stage linear mixed-effects meta-analysis to model the dose-response associations [29]. This method estimates the study-specific slope lines and combines them to obtain an average slope in a single stage. It includes studies with two categories of exposures in the dose-response analysis.

## 3. Results

### 3.1. Study Characteristics

**Figure 1:**
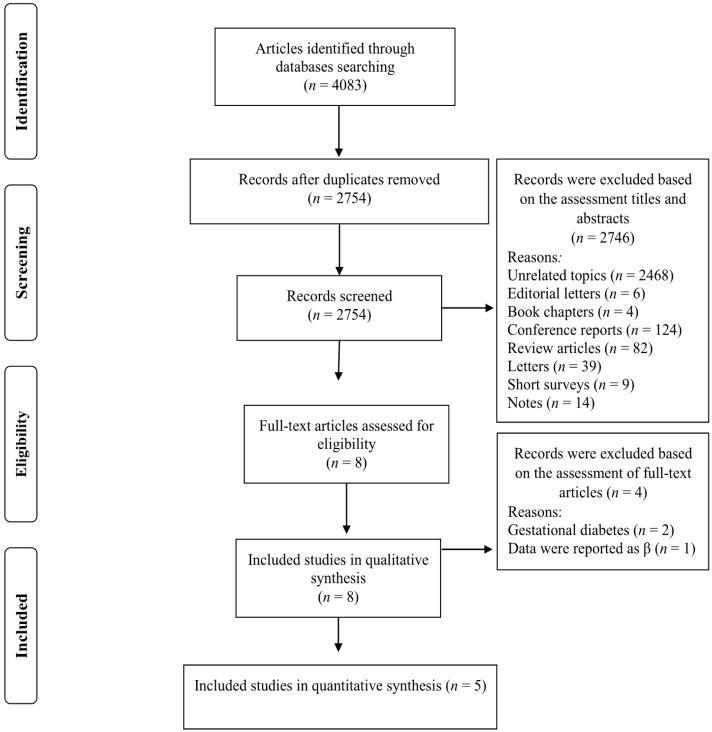
Flow chart of the process of the study selection.

Our search strategy retrieved 4083 studies and, after removing duplicates records, 2754 studies remained. The titles and abstracts of these studies were assessed and 2745 records were subsequently excluded based on our inclusion criteria. Eight full-text articles were reviewed and three of these studies were omitted due to the following reasons: two studies were conducted on women with gestational diabetes [30,31] and one study reported relevant data as βeta coefficients [32] (Figure 1). Five studies met our inclusion criteria and were included in our meta-analysis [7,19,20,21,22]. The characteristics of the included studies are summarized in Table 1 and described below. Of the five included studies, four were prospective cohort study designs [7,19,20,22] and the other was cross-sectional [21]. The follow-up duration for the cohort studies ranged from 3.4 to 12.0 years. The selected studies were published between 2019 and 2021 and were carried out in France [19], Spain [22], Netherlands [20], United Kingdom [7] and Canada [21]. The study-specific, maximally adjusted OR, HR, or RR were extracted across the selected studies and were pooled for meta-analysis to evaluate the association between UPF consumption and the risk of T2DM in a total of 230,526 adults. The quality of studies assessed via the Newcastle–Ottawa Scale indicated that all of the included articles had high quality (≥8 stars) (Table 1).

### 3.2. Ultra-Processed Food Consumption and T2DM Risk

**Figure 2:**
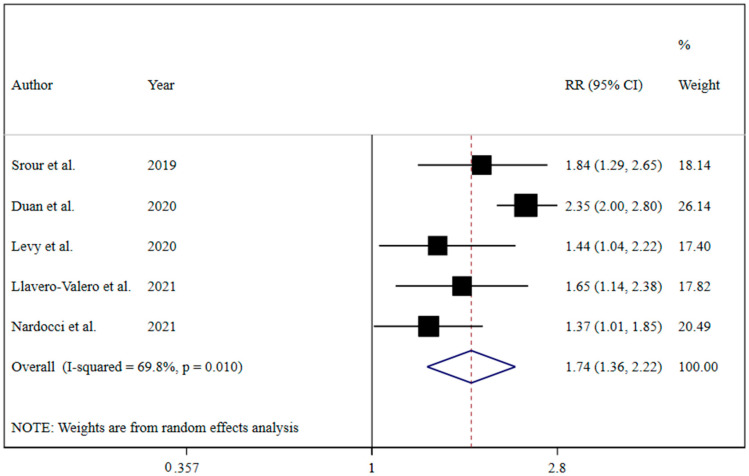
Forest plots demonstrating OR and 95% CI of pooled results from the random-effects models to evaluate the relationship between ultra-processed food consumption and risk of type 2 diabetes [7,19,20,21,22].

**Figure 3:**
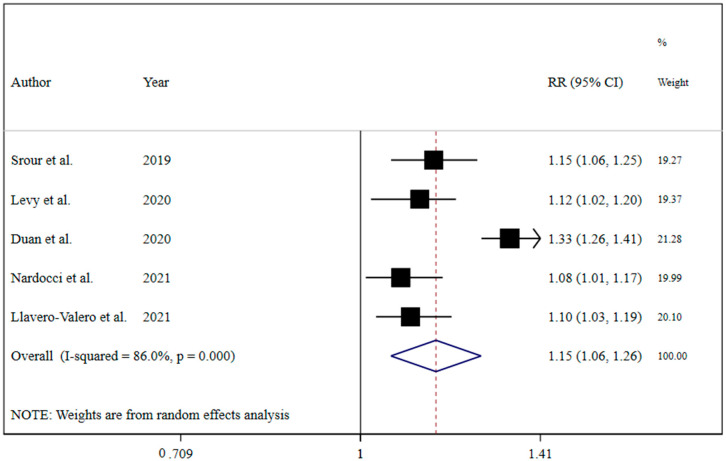
Forest plots showing the linear dose-response meta-analysis of type 2 diabetes risk for each 10% increase in ultra-processed food consumption in daily calorie intake [7,19,20,21,22].

**Figure 4:**
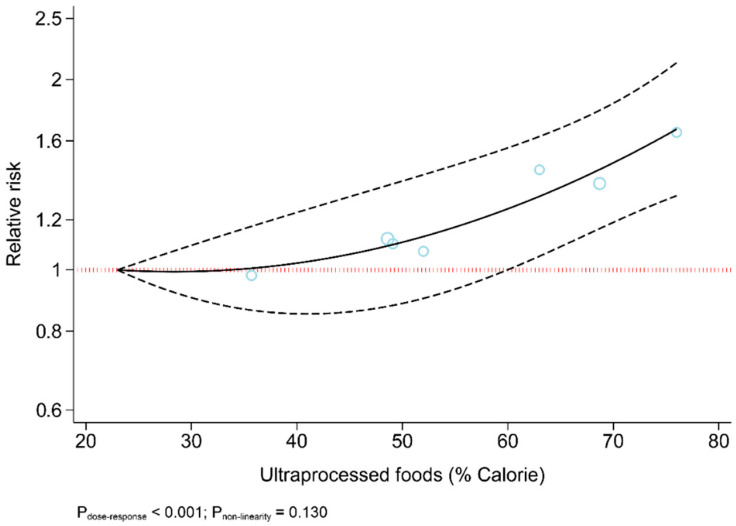
Dose-response association between ultra-processed food consumption and risk of type 2 diabetes.

The results revealed that UPF consumption was associated with an increased risk of T2DM (RR = 1.74; 95% CI: 1.36, 2.22; I^2^ = 68.9%; *p* < 0.001) (Figure 2). Linear dose-response analysis indicated that each 10% increase in UPF consumption was associated with a 15% higher risk of T2DM (RR = 1.15; 95% CI: 1.06, 1.26; I^2^ = 86.0%; *p* < 0.001) among adults (Figure 3). Moreover, non-linear dose-response associations are shown in Figure 4. This analysis demonstrated a positive linear association between UPF consumption with T2DM (*p*_nonlinearity_ = 0.13, *p*_dose-response_ ≤ 0.001) among adults.

### 3.3. Sensitivity Analyses

Sensitivity analysis showed that the study results were not affected by any single study.

### 3.4. Publication Bias

**Figure 5:**
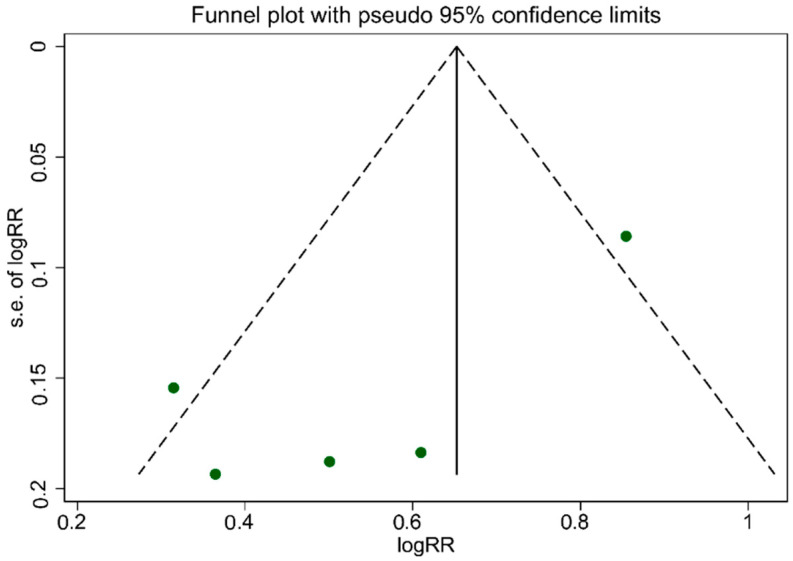
Funnel plot for evaluation publication bias.

As illustrated in Figure 5, the funnel plot was asymmetrical, which indicated publication bias. The evidence of publication bias among studies associated with UPF consumption, which is related to an increased risk of T2DM, was confirmed according to Egger’s regression asymmetry (*p* = 0.007). However, Begg’s rank correlation tests (*p* = 0.463) result did not show publication bias among studies.

## 4. Discussion

This study demonstrated that higher UPF intake was associated with an increased risk of T2DM. Additionally, increasing the intake of processed foods by 10% leads to a 15% increase in the risk of T2DM. A linear positive association was found between UPF intake and the risk of T2DM. The results of each study included in our meta-analyses were in line with our findings; they each showed that increased UPF intake was associated with an increased odds of the prevalence or incidence of T2DM. Two cohort studies conducted in Netherlands and Brazil reported a 10% increase in UPF consumption was associated with a 33% and 13% enhanced risk of developing T2DM, respectively. The other two cohort studies conducted in Spain and the United Kingdom showed that participants who consumed a higher versus a lower amount of UPF had a 53% and 44% higher risk of developing T2DM [7,22]. The last study, which was cross-sectional by design and conducted in Canada, demonstrated that participants with higher versus lower UPF intakes had 37% higher odds of T2DM [21].

Our study adds to the body of literature showing that particular dietary factors such as those common to UPF may foster certain aberrant health states, including diabetes. These dietary factors mainly include low nutritional quality, which more specifically consists of higher intakes of sodium, energy, fat, sugar, lower amounts of fiber, protein, essential vitamins and minerals. Indeed, our findings are consistent with a recent meta-analysis of observational studies that showed individuals with diabetes had higher sodium status compared to individuals without diabetes [33]. The authors hypothesized that underlying mechanisms for this association might be linked with the notion that excessive salt intake (>2.3 g/d) may activate the aldose reductase–fructokinase pathway in the hypothalamus and liver [33]. This activation of the aldose reductase–fructokinase pathway may lead to increased endogenous production of fructose and enhanced leptin resistance, both of which are implicated in insulin resistance [34].

The typically scarce levels and limited types of dietary fiber in UPF may also play a role in the observed association between UPF intake and T2DM. Dietary fiber is often recommended as adjunctive nutritional therapy in diabetes management. This is because dietary fiber lowers postprandial hyperglycemia and increases satiety by delaying digestion and absorption of carbohydrates and improving blood lipids, body mass and inflammation [35]. In addition, dietary fiber may increase peripheral insulin sensitivity through short-chain fatty acids produced by the gut microbiota and their capacity to ferment fiber [36,37]. Some studies also demonstrated that UPFs might directly modulate gut microbiota configuration, richness and diversity [38,39].

Another possible mechanism relevant to the dietary properties of UPF that may partly explain the association between UPF intake and T2DM is the added sugar content of UPF. A study conducted in the United States showed that almost 90% of the added sugar consumed by people is obtained from UPF [40]. It is widely accepted that excess sugar consumption can indirectly promote insulin resistance and diabetes by increasing body mass. Refined sugars common to UPF such as fructose and sucrose may also not be easily absorbed and metabolized by the liver, leading to increased lipid accumulation in the liver and decreased insulin sensitivity [41]. Fructose may also increase inflammatory responses that have been evidenced in impaired hepatic insulin signaling [42]. Furthermore, UPF may affect the glycemic response of these foods, with UPF being linked with a higher glycemic response compared to less processed or minimally processed foods [43].

While nutrition research has historically targeted the role of dietary calorie intake and macro and micronutrients in T2DM, the relevance of the NOVA food classification is underscored when considering non-nutritional factors related to food processing. These include incorporating artificial additives into UPF and the manifestation and/or migration of chemical compounds that may occur due to the production process and the encasing of formulations with packaging made from synthetic materials, respectively. For example, food additives such as carrageenan as a thickener and stabilizer can interfere with insulin signaling and may cultivate insulin resistance [44]. UPF is also often packaged in synthetic substances that may be a source of endocrine hormone-disrupting chemicals, including bisphenol A (BPA) and phthalates, both of which are associated with diabetes [45]. More specifically, BPA may promote T2DM through several pathways, including increased body mass, insulin resistance, inflammation and oxidative stress, as well as impaired glucose homeostasis and disrupted beta-cell function [46]. A recent meta-analysis study showed a positive, significant association between exposure to chemical compounds commonly used in industrial processes and food packaging, such as phthalates and insulin resistance [47]. Other common neoformed contaminants associated with food ultra-processing include acrolein, acrylamide and polycyclic aromatic hydrocarbons (PAH). Cooking fat and starchy foods at high temperatures produce acrolein (an unsaturated aldehyde) and acrylamide, respectively [48]. Acrolein and Acrylamide exposure have been linked with an increased risk of insulin resistance and diabetes and cardiovascular disease [49,50,51]. Exposure to PAH may cause oxidative stress and inflammation implicated in the pathogenesis of insulin resistance and beta-cell dysfunction [52,53]. Indeed, a study conducted in National Health And Nutrition Examination Survey (for the years 2005–2014) showed a positive, cross-sectional association between PAH exposure and diabetes prevalence [54].

While the high quality of studies included in our review, as per the NOS that was applied, may indicate reliable evidence, results from our meta-analyses should be viewed considering the following limitations. Few original studies were included in our syntheses, where inadequate estimation of the between-study variance remains possible [55]. In addition, dietary records of individuals at the time of measurement in the cross-sectional study may not represent habitual dietary intake. However, the inclusion of four other longitudinal studies, particularly the repeated dietary measurements after ten years of follow-up utilized by the prospective SUN cohort study in Spain [22], with identical findings, suggest robust results. In addition, information bias cannot be ruled out considering dietary intake data were self-reported and given that the dietary intake tools used by the original studies were not developed to identify UPF. While the longitudinal studies included in our review underscore a temporal order of the observed associations that moves from the intake of UPF to the subsequent development of T2DM, the observational nature of these studies means that causation cannot be determined and residual confounding not be eliminated. Another limitation that may partly explain the high heterogeneity, as per the I^2^ > 50% for our meta-analyses, was the between-study difference in expressing UPF intake as the exposure variable. For example, UPF intake was expressed as a continuous-only variable in two studies (10% increments) [19,20] compared to a categorical variable in the other three studies, which were also defined by the original study samples rather than a predetermined cut-off point (tertiles [21,22] and quartiles [7]). UPF intake was also calculated as either absolute intake or a proportion (%) per day of energy (kilocalorie) [21] or weight (grams) [7,19,22], which may have contributed to the high heterogeneity. Moreover, the identifications of T2DM incidents and ultra-processed food intake among included studies were according to self-reported or nurse interview reports. Self-reported records are usually limited by underreporting ultra-processed food intake and misclassification of subjects resulting in a potential bias in the under-finding of T2DM cases. Ultimately, the ultra-processed classification includes various products; this exploratory strategy was not intended to focus on a particular food class or isolate a specific process/additive. Nonetheless, it let us examine general exposure to UPF and observe relations with T2DM resulting from cumulative intakes and possible effects of their components.

## 5. Conclusions

The current systematic review and dose-response meta-analysis was conducted to elucidate whether the UPF consumption has a potential association with the risk of T2DM. Observational studies undertaken in adults that met inclusion criteria were included in the final analysis. In summary, our outcomes suggested that higher UPF consumption may be significantly associated with an increased risk of T2DM. In addition, the linear dose-response analysis showed that each 10% increase in UPF consumption (kcal/d) was associated with a 15% higher risk of T2DM among adults. Non-linear dose-response analysis also demonstrated a positive linear association between UPF consumption and T2DM among adults. In terms of directions for future research, further studies could investigate the following points. Current tools available for estimating UPF intakes are subjective (food records, FFQ, 24 h recalls, diet history with interviewer-assisted data collection), rather limited in scope, with a majority evaluating only one dimension (i.e., cumulative UPF intakes). To more precisely estimate the actual burden of UPF intakes, new tools should be adapted or progressed to evaluate all UPF consumption dimensions, namely food class, UPF foods specific components, their effects on health and specific processes or additives. The new technology-based dietary assessment methods (web-based and mobile device applications) may help more precise UPF intakes evaluation. However, underlying mechanisms remain unknown. In future studies, evaluation of associated variables including lifestyle, demographic, genetic background, socioeconomic and clinical factors, as well as differences in treatment, can accelerate the finding of possible mechanisms. Due to identified limitations, to better understand the association between UPF consumption and the risk of T2DM, more prospective cohort studies with further extended follow-up periods are necessary.

## Figures and Tables

**Table 1:** Characteristics of the included studies.

Author (Year; Location)	Study Design/Follow Up	Population/	Ultra-Processed Food Assessment Method	Outcomes	Adjusted Variables	Quality Score
	(Years)/Source of Data/Health Status	Age/(Women/Men)/Sex				
Srour et al. (2019, France) [19]	Prospective Cohort/6 years/the French NutriNet-Santé cohort (2009–2019)/Healthy subjects	N = 104,707/	24 h food records/NOVA food classification/Proportion of weight	Absolute increment of 10% of UPF in the diet increases the risk of developing T2D, (HR = 1.13; 95% CI: 1.01, 1.27)	Age, sex, educational level, BMI, physical activity, smoking, alcohol, number of 24-h dietary records, energy intake, family history of T2D, FSAm-NPS DI score, percentage of weight change among participants with available repeated anthropometric data	0.9
		Age ≥ 18 years/				
		(82,907/21,800)				
Duan et al. (2020, The Netherlands) [20]	Population-based cohort/3.4 years/Lifelines cohort study/Healthy subjects	N = 70421/	FFQ/NOVA foodclassification/Proportion of weight	Increment of 10% of UPF in the diet increases the risk of developing T2D (OR = 1.33; 95% CI: 1.26, 3.141)	Adjustments for confounders, including overall diet quality.	0.8
		Age = 30–70 years/				
		(41,267/29,154)				
Levy et al. (2020, United Kingdom) [7]	Prospective Cohort/5.4 years/the UK Biobank (2007–2019)/Healthy subjects	N = 21,730/	24 h food records/NOVA food classification/Proportion of weight	Adults in the highest quartile	Age, family history of T2D, stratification by sex and ethnicity, index of multiple deprivation, physical activity level, current smoking status, total energy intake, and BMI continuous at baseline.	0.8
		Age ≥ 18 years/		of UPF consumption had 44% higher odds of T2D (HR = 1.44, 95% CI: 1.04–2.02)		
		(11,299/10,431)				
Nardocci et al. (2021, Canada) [21]	Cross-sectional/-/Canadian Community Health Survey–Nutrition/Diabetes and hypertension subjects	N = 13,608/	24 h recalls/NOVA food classification/Proportion of daily energy intake	Adults in the highest tertile	Age, sex, smoking status, physical activity, alcohol consumption, education, income, residential area, immigrant status, Indigenous identity.	0.8
		Age ≥ 19 years/(6801/6807)		of UPF consumption had 37% higher odds of T2D (OR = 1.37, 95% CI: 1.01–1.85)		
Llavero-Valero et al. (2021, Spain) [22]	Prospective cohort/12 years/the SUN project/Healthy subjects	N = 20,060/	FFQ/NOVA food classification/Proportion of weight	Adults in the highest tertile of UPF consumption had 53% higher odds of T2D (HR = 1.53, 95% CI: 1.06–2.22)	Age, tertiles of BMI, educational status, family history of diabetes, smoking status, snacking between meals, 8-item active þ sedentary lifestyle score and following a special diet at baseline. Stratified by decades of age and recruitment period	0.9
		Age ≥ 18 years/(8218/11,842)				

Abbreviations: UPF: Ultra-processed foods; T2D: Type 2 diabetes; HR: Hazard ratio; FFQ: Food Frequency Questionnaires, BMI: Body mass index; FSAm-NPS DI: Food Standard Agency nutrient profiling system, dietary index; OR: Odds ratio; SUN: Seguimiento Universidad de Navarra.

## Supplementary Materials

The following are available online at https://www.mdpi.com/article/10.3390/nu13124410/s1, Table S1: Description of population, intervention, comparator and outcome (PICO). Table S2: Search strategies including the key terms and the queries for each database.
