# Supplementary material for: Ultra-Processed Food Consumption and Adult Diabetes Risk: A Systematic Review and Dose-Response Meta-Analysis
Source: Nutrients. 2021 Dec 9;13(12):4410. doi: 10.3390/nu13124410 (PMC8705763; doi:10.3390/nu13124410)
Supplement: Supplementary file 1 [file nutrients-13-04410-s001.zip › Supplementary Table S1.pdf]

Supplementary Table S1. Description of population, intervention, comparator and outcome (PICO).

**Population**	Adults (≥18 years)
**Intervention**	-
**Comparison**	Ultra-processed Food Consumption (higher versus lower)
**Outcome**	Diabetes Risk
