# Supplementary material for: Ultra-Processed Food Consumption and Adult Diabetes Risk: A Systematic Review and Dose-Response Meta-Analysis
Source: Nutrients. 2021 Dec 9;13(12):4410. doi: 10.3390/nu13124410 (PMC8705763; doi:10.3390/nu13124410)
Supplement: Supplementary file 1 [file nutrients-13-04410-s001.zip › Supplementary Table S2.pdf]

Supplementary Table S2. Search strategies including the key terms and the queries for each database

**Database 10/8/2021**	**key terms and the queries**
PubMed (n=1477)	#1 "fast foods"[All Fields] OR "fast foods"[MeSH Terms] OR "ultra processed food*"[All Fields] OR "ultraprocessed food*"[All Fields] OR "ultra processed food*"[All Fields] OR "processed food*"[All Fields] OR "ultra-processed"[All Fields] OR "ultraprocessed"[All Fields] OR "ultra-processed"[All Fields] OR "NOVA"[All Fields] OR "nova food classif*"[All Fields] OR "nova food*"[All Fields] OR "nova food classif*"[All Fields] OR "NOVA food classification system"[All Fields]<br><br>#2 "Diabetes Mellitus"[MeSH Terms] OR "diabetes mellitus, type 2"[MeSH Terms] OR "Diabetes Mellitus"[All Fields] OR "diabetes"[All Fields] OR "T2DM"[All Fields] OR "type 2 diabetes mellitus"[All Fields]<br><br>#3 #1 AND #2
Web of Science (n=536)	#1 TOPIC: ("ultra-processed food*") OR TOPIC: ("ultraprocessed food*") OR TOPIC: ("ultra processed food*") OR TOPIC: ("processed food*") OR TOPIC: ("ultra processed") OR TOPIC: ("ultraprocessed") OR TOPIC: ("ultra-processed") OR TOPIC: ("NOVA") OR TOPIC: ("NOVA food classif*") OR TOPIC: ("NOVA food*") OR TOPIC: ("NOVA food classif*") OR TOPIC: ("NOVA food classification system") OR TOPIC: ("fast foods")<br><br>#2 TOPIC: ("Diabetes Mellitus") OR TOPIC: ("diabetes") OR TOPIC: ("T2DM") OR TOPIC: ("type 2 diabetes mellitus")<br><br>#3 #1 AND #2
Scopus (n=2321)	#1 (TITLE-ABS-KEY ("ultra-processed food*") OR TITLE-ABS-KEY ("ultraprocessed food*") OR TITLE-ABS-KEY ("ultra processed food*") OR TITLE-ABS-KEY ("processed food*") OR TITLE-ABS-KEY ("ultra processed") OR TITLE-ABS-KEY ("ultraprocessed") OR TITLE-ABS-KEY ("ultra-processed") OR TITLE-ABS-KEY ("NOVA") OR TITLE-ABS-KEY ("NOVA food classif*") OR TITLE-ABS-KEY ("NOVA food*") OR TITLE-ABS-KEY ("NOVA food classif*") OR TITLE-ABS-KEY ("NOVA food classification system") OR TITLE-ABS-KEY ("fast foods"))<br><br>#2 TITLE-ABS-KEY ("Diabetes Mellitus") OR TITLE-ABS-KEY ("diabetes") OR TITLE-ABS-KEY ("T2DM") OR TITLE-ABS-KEY ("type 2 diabetes mellitus")<br><br>#3 #1 AND #2
